# Precision Medicine in Hematologic Malignancies: Evolving Concepts and Clinical Applications

**DOI:** 10.3390/biomedicines13071654

**Published:** 2025-07-07

**Authors:** Rita Khoury, Chris Raffoul, Christina Khater, Colette Hanna

**Affiliations:** 1 Division of Hematology/Oncology, Lebanese American University Medical Center-Rizk Hospital, Beirut 1100, Lebanon; rita.khoury011@gmail.com (R.K.); christina.khater01@lau.edu.lb (C.K.); colette.hanna@lau.edu.lb (C.H.); 2 Department of Internal Medicine at VCU Health, Richmond, VA 23298, USA

**Keywords:** precision medicine, hematologic malignancies, genomic profiling, targeted therapy, liquid biopsy, minimal residual disease, immunotherapy, artificial intelligence

## Abstract

Precision medicine is transforming hematologic cancer care by tailoring treatments to individual patient profiles and moving beyond the traditional “one-size-fits-all” model. This review outlines foundational technologies, disease-specific advances, and emerging directions in precision hematology. The field is enabled by molecular profiling techniques, including next-generation sequencing (NGS), whole-exome sequencing (WES), and RNA sequencing (RNA-seq), as well as epigenomic and proteomic analyses. Complementary tools such as liquid biopsy and minimal residual disease (MRD) monitoring have improved diagnosis, risk stratification, and therapeutic decision making. We discuss major molecular targets and personalized strategies across hematologic malignancies: *FLT3* and *IDH1/2* in acute myeloid leukemia (AML); Philadelphia chromosome–positive and Ph-like subtypes in acute lymphoblastic leukemia (ALL); *BCR-ABL1* in chronic myeloid leukemia (CML); *TP53* and *IGHV* mutations in chronic lymphocytic leukemia (CLL); molecular subtypes and immune targets in diffuse large B-cell lymphoma (DLBCL) and other lymphomas; and B-cell maturation antigen (BCMA) in multiple myeloma. Despite significant progress, challenges remain, including high costs, disparities in access, a lack of standardization, and integration barriers in clinical practice. However, advances in single-cell sequencing, spatial transcriptomics, drug repurposing, immunotherapies, pan-cancer trials, precision prevention, and AI-guided algorithms offer promising avenues to refine treatment and improve outcomes. Overcoming these barriers will be critical for ensuring the equitable and widespread implementation of precision medicine in routine hematologic oncology care.

## 1. Introduction

Precision medicine refers to tailoring medical treatment based on an individual’s genetic, molecular, and environmental factors. In oncology, and particularly in hematologic malignancies, this approach involves reshaping diagnosis, risk assessment, and therapeutic decision making [1,2]. Hematologic cancers account for an estimated 1.3 million new cases and over 700,000 deaths globally each year, with non-Hodgkin lymphoma, leukemia, and multiple myeloma being the most common and deadly subtypes [3]. These malignancies are driven by distinct molecular alterations, leading to diverse subtypes and varying treatment responses. This complexity makes them ideal candidates for personalized treatment strategies [1,2].

Traditionally, hematologic malignancies have been treated with a “one-size-fits-all” approach based on broad diagnostic categories, which often neglects individual variations in disease biology. These standardized protocols can result in inconsistent therapeutic outcomes. Precision medicine seeks to overcome this by aligning treatment with the unique genetic profile of each patient’s disease [4,5].

Recent advances in genomic profiling, epigenomic and proteomic analyses, and liquid biopsy technologies have dramatically improved our understanding of tumor biology. These tools help identify actionable mutations, refine disease classification, and guide the selection of targeted therapies [6,7,8,9]. Genomic testing, for example, enables precise molecular subtyping for diagnosis, while specific mutations and gene signatures aid in risk stratification [10]. Treatment selection is increasingly driven by molecular markers, with immunotherapies and targeted agents tailored to the individual profile [11]. Monitoring techniques have also evolved, with minimal residual disease (MRD) detection and circulating tumor DNA (ctDNA) analysis offering more sensitive, non-invasive monitoring of treatment response and disease progression [12,13].

This review provides a comprehensive overview of precision medicine in hematologic cancers, highlighting key technologies, disease-specific applications, and future directions for integrating molecular insights into routine clinical practice.

## 2. Technological Foundations of Precision Hematology

The integration of molecular and genomic data has revolutionized hematology, enabling more precise diagnoses, better risk predictions, and more tailored treatments [1,2]. Central to this shift are three main technological pillars: genomic profiling, including next-generation sequencing (NGS), whole-exome sequencing (WES), and RNA sequencing (RNA-seq); epigenomic and proteomic analyses; and liquid biopsy technologies, especially the use of ctDNA for disease monitoring, including MRD assessment [6,7,8].

These technologies help clinicians understand the genetic and molecular landscape of hematologic cancers, offering deeper insights into disease biology and supporting personalized treatment strategies [1,2,6,7,8].

### 2.1. Genomic Profiling Tools

#### 2.1.1. Next-Generation Sequencing (NGS)

NGS is a high-throughput technology that allows for the simultaneous analysis of millions of DNA and RNA fragments, detecting genomic abnormalities such as single-nucleotide variants (SNVs), insertions and deletions (indels), copy number variations (CNVs), and chromosomal translocations [14,15]. Depending on the clinical need, NGS can be performed using targeted panels that focus on specific genes of interest or through more comprehensive approaches like whole-genome sequencing (WGS). Compared to traditional methods like Polymerase Chain Reaction (PCR) and Sanger sequencing, NGS provides a much broader genomic analysis in a single test, making it invaluable for both research and clinical applications in cancer [10,14,16].

In hematologic malignancies, NGS plays a crucial role in defining the genetic landscape, identifying mutations that assist in diagnosis, risk stratification, and treatment planning [2,10,17]. NGS also helps select targeted therapies by detecting actionable mutations and resistance mutations, which can guide therapy adjustments [5,11,17]. Additionally, NGS is an essential tool for monitoring MRD, as its high sensitivity enables the detection of low levels of residual cancer cells, providing early insights into relapse risk [18].

However, NGS faces challenges such as cost, the complexity of data interpretation, bioinformatics hurdles, and the need for specialized expertise in clinical settings. The large volume of data generated requires expert analysis, and the lack of standardization in assay design and reporting across laboratories can lead to reproducibility issues [19,20,21]. Moreover, incidental findings—mutations unrelated to the primary disease—raise ethical concerns about disclosure and management [22]. Despite these challenges, NGS remains a cornerstone of precision medicine in hematologic cancers [10].

#### 2.1.2. Whole-Exome Sequencing (WES)

WES targets the protein-coding regions of the genome, which account for about 1% of the total genome but harbor the majority of known disease-causing mutations. Unlike WGS, which covers both coding and non-coding regions, WES offers a more focused and cost-effective approach for detecting clinically relevant mutations [23]. WES is especially useful in cases where the diagnosis remains unclear, such as in undiagnosed conditions, inherited cancer syndromes, and pediatric malignancies [24,25]. It plays a crucial role in uncovering germline predisposition syndromes, such as Li–Fraumeni syndrome (TP53 mutations) and GATA2 deficiency, that may not be detected through standard diagnostic methods and can have significant implications for familial risk assessment and long-term management [26,27,28]. However, WES generally has a lower sequencing depth than targeted panels, potentially missing low-frequency variants, and it does not capture non-coding regions, which can harbor disease-relevant mutations [29,30].

#### 2.1.3. RNA Sequencing (RNA-Seq)

RNA-seq analyzes gene expression and detects fusion transcripts, providing insights into the functional state of cancer cells [31]. It is crucial for identifying fusions like BCR-ABL and for uncovering alternative splicing events and dysregulated signaling pathways in leukemias and lymphomas [31,32]. RNA-seq has gained increasing clinical relevance as it characterizes disease biology and informs treatment decisions, particularly in rare or ambiguous cases [32]. However, RNA is inherently unstable and prone to degradation, making RNA-seq highly sensitive to RNA quality and sample handling conditions [33].

#### 2.1.4. Comparative Overview of Genomic Profiling Approaches

To summarize the key distinctions between genomic tools discussed above, Table 1 highlights their main features, advantages, and limitations, as described in this section.

While genomic tools like NGS, WES, and RNA-seq uncover mutations and structural variations, complementary approaches such as epigenetic and proteomic profiling provide insights into gene regulation and protein-level alterations, enhancing our understanding of cancer biology.

### 2.2. Epigenomics and Proteomics

#### 2.2.1. Epigenomics

Epigenomics explores gene regulation changes that do not involve alterations in the DNA sequence itself. DNA methylation, one of the best-characterized mechanisms, plays an important role in regulating gene expression. In hematologic malignancies like acute myeloid leukemia (AML) and myelodysplastic syndrome (MDS), abnormal methylation patterns are frequently seen and are key contributors to disease development [8,34].

Technologies such as methylation arrays and bisulfite sequencing enable the high-resolution mapping of these changes, helping to define epigenetic signatures for specific cancers. For instance, AML cases with *IDH* mutations often exhibit hypermethylation due to the inhibition of DNA demethylation enzymes by 2-hydroxyglutarate [35,36,37].

Epigenomic profiling has emerging clinical relevance, particularly in refining risk stratification through methylation-based biomarkers [8,38]. Interest in epigenetically targeted therapies is growing, with hypomethylating agents and *IDH* inhibitors demonstrating how the modulation of epigenetic alterations can support precision medicine. Azacitidine and decitabine—both DNA methyltransferase inhibitors—are examples of hypomethylating agents already used in clinical practice, especially in MDS, where they help reverse abnormal gene silencing. These advances highlight the role of epigenomics in uncovering disease mechanisms and enabling more personalized treatment strategies [8,36].

#### 2.2.2. Proteomics

Proteomics involves the large-scale study of proteins, offering insights into disease biology by examining protein expression, structure, interactions, and post-translational modifications that can alter protein function or activity [39]. For example, the phosphorylation of *FLT3* is a key event in leukemogenesis and is used to monitor signaling activity in AML [40]. This approach provides a direct view of cancer mechanisms that may not be captured by genomics. Techniques like mass spectrometry and immunoassays enable precise protein quantification, allowing for the identification of disease-specific biomarkers and the monitoring of signaling pathways [39,41].

Proteomics, when integrated with genomic and epigenomic data, can enhance cancer diagnosis, prognosis, and treatment monitoring. For example, CD123 has been identified as a biomarker for blastic plasmacytoid dendritic cell neoplasm (BPDCN), and it is also an emerging therapeutic target, with agents like tagraxofusp (a CD123-directed antibody) demonstrating clinical activity [9,42]. Meanwhile, phosphoproteomics is used to monitor signaling pathways in other cancers [43].

Beyond molecular profiling, dynamic monitoring tools such as liquid biopsy and MRD assessment provide real-time insights into disease burden and therapeutic response, enabling more precise and timely clinical decision making.

### 2.3. Liquid Biopsy and Minimal Residual Disease Monitoring

#### 2.3.1. Circulating Tumor DNA (ctDNA)

ctDNA refers to tumor-derived DNA fragments in the bloodstream, offering a non-invasive way to monitor disease via blood samples. ctDNA analysis allows for the real-time assessment of tumor genetics through techniques like PCR and NGS of plasma circulating free DNA (cfDNA). It is widely used to monitor treatment response, detect resistance mutations, and assess relapse risk. Serial sampling can enable the early detection of molecular relapse and timely interventions, making ctDNA a powerful tool for personalized care [13,44]. However, a current limitation is that ctDNA levels may be low or undetectable in some malignancies, potentially resulting in false-negative results and reduced sensitivity, thereby limiting its clinical utility in these settings [45].

#### 2.3.2. Minimal Residual Disease (MRD) Monitoring

MRD monitoring detects small amounts of residual disease after treatment, offering insight into relapse risk. Techniques such as flow cytometry, PCR, and NGS can identify these low levels of disease, even when clinically undetectable [12]. MRD status is a strong prognostic indicator, with MRD-negative patients showing better survival rates and lower relapse risks [12,46]. As a result, MRD monitoring plays a crucial role in guiding treatment decisions, such as therapy escalation or de-escalation, tailored to the patient’s disease burden [47]. Major guidelines such as those from the National Comprehensive Cancer Network (NCCN) and the European LeukemiaNet (ELN) increasingly support MRD-guided therapeutic strategies in diseases like acute lymphoblastic leukemia (ALL) and AML, highlighting its growing clinical relevance [48,49]. Limitations include challenges in assay standardization, differences in method sensitivity, and limited access in some settings [12].

By integrating genomic, epigenomic, proteomic, and liquid biopsy tools, clinicians can enhance diagnostic accuracy, improve risk stratification, select targeted therapies, and monitor disease. These technologies are becoming part of routine hematology practice and reflect the growing translation of molecular tools into clinical care, redefining disease classification and treatment strategies [7,8,38,44].

**Figure 1:**
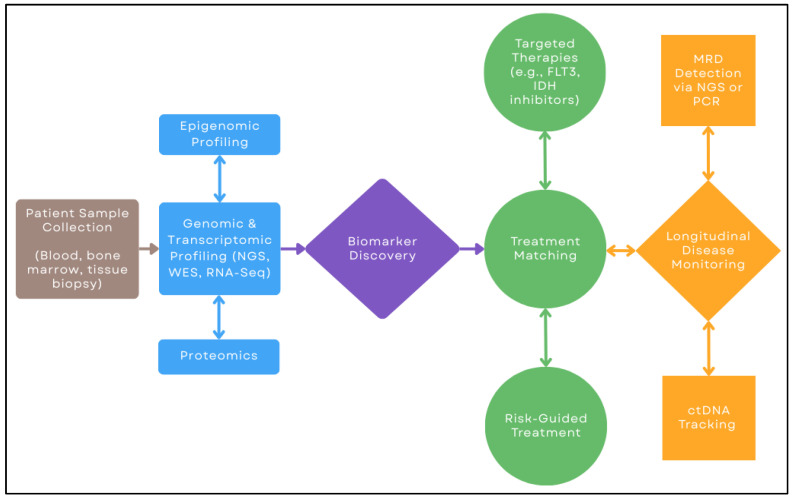
Workflow of precision hematology: from patient sample collection to biomarker identification, treatment matching, and longitudinal disease monitoring. NGS: next-generation sequencing; WES: whole-exome sequencing; RNA-seq: RNA sequencing; MRD: minimal residual disease; PCR: Polymerase Chain Reaction; ctDNA: circulating tumor DNA.

Before diving into disease-specific advances, Figure 1 summarizes the general workflow of precision hematology, from genomic profiling to treatment selection and ongoing monitoring.

## 3. Disease-Specific Advances in Precision Medicine

Precision medicine has transformed the diagnosis, risk stratification, and treatment of hematologic malignancies. Disease-specific molecular features now influence therapeutic decisions, enabling more personalized care. The following sections highlight key molecular targets as well as diagnostic and therapeutic advancements in major hematologic cancers.

### 3.1. Acute Myeloid Leukemia

AML is a heterogeneous disease characterized by diverse molecular alterations that impact prognosis and treatment response. Precision medicine in AML focuses on identifying recurrent mutations such as *FLT3*, *IDH1/2*, *NPM1*, and *TP53* to guide therapeutic decisions [50].

*FLT3* mutations, found in approximately 30% of AML cases, particularly the internal tandem duplication (*FLT3-ITD*), are associated with poor prognosis [51]. *FLT3* inhibitors such as midostaurin, gilteritinib, and more recently quizartinib have improved outcomes, especially when combined with chemotherapy in newly diagnosed patients or as monotherapy in relapsed/refractory settings [52,53,54]. However, resistance to *FLT3* inhibitors frequently emerges through secondary kinase domain mutations or the activation of alternative signaling pathways, limiting their long-term effectiveness and prompting ongoing investigation into optimal combination strategies [55].

*IDH1* and *IDH2* mutations contribute to leukemia development via epigenetic dysregulation [56]. Targeted *IDH* inhibitors—ivosidenib (*IDH1*) and enasidenib (*IDH2*)—have shown significant clinical benefit, especially in relapsed or refractory AML [57,58]. Nevertheless, response rates with *IDH* inhibitors remain modest, and not all patients with *IDH* mutations derive benefit, highlighting the need for synergistic treatment combinations [59].

*NPM1* mutations, present in about 30–35% of adult AML cases, are often associated with a favorable prognosis, particularly in the absence of *FLT3-ITD* mutations. They also serve as valuable biomarkers for both diagnosis and MRD monitoring using PCR [60]. Emerging strategies are now exploring menin inhibitors (e.g., revumenib, ziftomenib) in *NPM1*-mutated and *KMT2A*-rearranged AML, showing promising activity by disrupting oncogenic transcriptional programs [61].

Conversely, *TP53* mutations confer resistance to conventional chemotherapy and are linked to poor survival outcomes [62]. Although targeted treatments for *TP53*-mutant AML remain limited, hypomethylating agents such as azacitidine and decitabine, along with investigational drugs like APR-246 (eprenetapopt), have been explored in clinical trials, showing promising responses, particularly when used in combination [62,63,64].

Comprehensive genomic profiling is important not only for risk stratification and clinical trial eligibility but also for detecting clonal evolution and guiding therapy adaptation over time. This highlights the important role of precision medicine in modern AML management [50].

### 3.2. Acute Lymphoblastic Leukemia

In ALL, precision medicine has improved survival by identifying genetic subtypes and integrating the use of targeted immunotherapies [65].

Philadelphia chromosome-positive (Ph+) ALL, characterized by the *BCR-ABL1* fusion gene, has seen important advances through the incorporation of tyrosine kinase inhibitors (TKIs) such as imatinib and dasatinib, which are now standard components of therapy, as recommended by the NCCN [66,67,68]. In cases resistant to earlier-generation TKIs, asciminib—a novel STAMP inhibitor targeting the myristoyl pocket of *BCR-ABL1*—has emerged as a promising option and is currently under evaluation in Ph+ ALL [69]. However, resistance mutations such as *T315I* continue to pose challenges, and ponatinib, though effective against *T315I*, is associated with vascular toxicity at higher doses—leading to ongoing debates regarding dose optimization and risk–benefit balance [70,71]. Another high-risk subtype, Philadelphia-like (Ph-like) ALL, lacks the *BCR-ABL1* fusion but shares a similar gene expression profile and often harbors targetable kinase alterations involving ABL-class fusions or the JAK-STAT pathway [72]. This has led to the use of TKIs such as olverembatinib in selected patients based on underlying mutations [73,74].

Other targeted immunotherapies, such as blinatumomab—a bispecific T-cell engager (BiTE) targeting CD19—and inotuzumab ozogamicin—an anti-CD22 antibody–drug conjugate (ADC)—have further expanded treatment options in B-cell ALL, particularly in relapsed/refractory cases [75,76]. Chimeric antigen receptor (CAR) T-cell therapy targeting CD19, such as tisagenlecleucel, has also reshaped the therapeutic landscape, offering the possibility of durable remission in these patients [66,77]. Another CAR T-cell product, KTE-X19 (brexucabtagene autoleucel), has received FDA approval for relapsed/refractory adult ALL and represents a significant addition to the immunotherapy arsenal [78]. Despite high response rates, CAR T-cell therapy is associated with severe toxicities such as cytokine release syndrome (CRS) and neurotoxicity, as well as high cost, which limit accessibility and widespread adoption [79,80]. Moreover, relapse due to antigen escape (e.g., CD19-negative relapse) remains a significant clinical hurdle [81].

Moreover, relapse due to antigen escape (e.g., CD19-negative relapse) remains a significant clinical hurdle. Additionally, MRD monitoring plays a critical role in ALL management, serving as a relapse predictor and guiding decisions around treatment intensification or de-escalation [66].

In T-cell ALL, investigational therapies including *NOTCH1* inhibitors, *CDK4/6* inhibitors, and *BCL-2* inhibitors like venetoclax and navitoclax are being studied, though no targeted agents are yet FDA-approved [82,83,84,85].

Together, these strategies reinforce the growing importance of precision medicine in tailoring therapy and improving outcomes in ALL.

### 3.3. Chronic Myeloid Leukemia

Chronic myeloid leukemia (CML) represents a success in precision oncology, defined by the presence of the *BCR-ABL1* fusion gene, which encodes a constitutively active tyrosine kinase that drives malignant transformation [1,86]. The quantitative real-time PCR measurement of *BCR-ABL1* transcript levels is the standard method used for diagnosis, monitoring treatment response, and evaluating molecular remission [1].

The introduction of TKIs—including imatinib, dasatinib, nilotinib, bosutinib, and ponatinib—has redefined CML treatment and significantly improved survival outcomes [87]. However, resistance to TKIs can emerge, particularly due to mutations in the *BCR-ABL1* kinase domain [88]. Among these, the *T315I* mutation is especially challenging, conferring resistance to most TKIs except ponatinib [89]. Therefore, mutation profiling is essential for selecting the appropriate TKI to overcome resistance [1]. Newer agents such as asciminib provide an alternative mechanism of inhibition and are effective in resistant cases [90]. Nevertheless, long-term head-to-head comparisons between asciminib and other TKIs are lacking, and its role in frontline therapy remains under investigation [91].

The precision-guided application of TKIs, combined with serial molecular monitoring, has shifted the management of CML from a fatal leukemia into a chronic, controllable disease. For patients who achieve deep molecular responses, treatment-free remission (TFR) is now an attainable goal, further highlighting the impact of individualized therapy in CML [92].

### 3.4. Chronic Lymphocytic Leukemia

Chronic lymphocytic leukemia (CLL) is a genetically diverse malignancy, with treatment strategies increasingly guided by molecular risk factors. Among these, TP53 mutations and deletion of 17p [*del(17p)*] are associated with resistance to traditional chemoimmunotherapy and confer a poor prognosis [93]. These patients are preferentially treated with BTK inhibitors like ibrutinib, acalabrutinib, or zanubrutinib, or with venetoclax combined with anti-CD20 agents like Obinutuzumab [94,95,96].

Another prognostic marker is the immunoglobulin heavy chain variable (IGHV) gene mutation status. Patients with unmutated IGHV tend to have a more aggressive disease, whereas those with mutated IGHV generally experience a more indolent clinical course [93]. Routine NGS panels enable the detection of other prognostically relevant mutations, including those in *NOTCH1*, *SF3B1*, and *BIRC3*, which could help guide future treatments as more targeted drugs are introduced [97,98,99].

In relapsed or refractory settings, newer agents such as the non-covalent BTK inhibitor pirtobrutinib are being introduced to overcome resistance to earlier-generation BTK inhibitors [100]. However, resistance to both BTK and BCL-2 inhibitors is increasingly observed in clinical practice, and data on optimal sequencing or retreatment strategies remain sparse. Ongoing clinical trials are expected to inform best practices [101].

As such, using molecular profiling in clinical practice has changed how CLL is treated, moving away from traditional chemoimmunotherapy toward more targeted treatments with improved efficacy and tolerability [102].

### 3.5. Lymphomas

Lymphomas are a diverse group of cancers, and advances in molecular testing and targeted therapies are transforming how they are treated [103].

Diffuse large B-cell lymphoma (DLBCL), the most common subtype, can be divided into germinal center B-cell-like (GCB) and activated B-cell-like (ABC) subtypes based on gene expression [104]. This classification guides treatment decisions, as targeted drugs like BTK inhibitors, such as ibrutinib, have shown effectiveness in the ABC subtype [105].

High-grade B-cell lymphomas with rearrangements in the *MYC*, *BCL2*, and/or *BCL6* genes—referred to as “double-hit” or “triple-hit” lymphomas—tend to be more aggressive and often require more intensive treatment strategies [106].

In Hodgkin lymphoma, immune checkpoint inhibitors such as nivolumab and pembrolizumab have significantly improved outcomes, particularly in patients with relapsed or refractory disease [107]. These therapies work by targeting PD-L1, a protein that is often overexpressed in Hodgkin–Reed–Sternberg cells due to chromosome 9p24.1 amplification [108]. Additional ADCs such as polatuzumab vedotin (anti-CD79b) are used in relapsed/refractory DLBCL [109].

New targeted therapies have expanded options for relapsed/refractory DLBCL, including the anti-CD19 monoclonal antibody tafasitamab combined with lenalidomide and selinexor, a selective inhibitor of nuclear export. While these agents are effective in subsets of patients, the absence of robust biomarkers to predict response and the potential for overlapping toxicities limit their broader application. Moreover, CAR T-cell therapies such as axi-cel, liso-cel, and tisa-cel have revolutionized treatment for aggressive B-cell lymphomas, offering durable remissions in many patients [110].

Thus, the use of molecular diagnostics, immunophenotyping, and targeted therapies continues to improve the precision and effectiveness of lymphoma treatment [103].

### 3.6. Multiple Myeloma

Multiple myeloma (MM) is a malignancy of plasma cells characterized by variable clinical behavior, influenced by genetic and cytogenetic abnormalities.

Risk stratification in MM relies on identifying specific markers through fluorescence in situ hybridization (FISH), including translocations such as *t(4;14*) and *t(14;16)*, as well as deletions like *del(17p).* Gene profiling is also used to refine prognosis and determine the treatment intensity needed for individual patients [111].

An important advance in MM therapy has been the identification of B-cell maturation antigen (BCMA) as a therapeutic target expressed on malignant plasma cells. Several novel BCMA-targeted treatments have been approved, including CAR T-cell therapies such as idecabtagene vicleucel and ciltacabtagene autoleucel, bispecific antibodies like teclistamab and elranatamab, and ADCs such as belantamab mafodotin [111,112]. Other key therapeutic advances include anti-CD38 monoclonal antibodies such as daratumumab and isatuximab, which have become the integral components of multiple myeloma treatment regimens. Emerging therapies also include cereblon E3 ligase modulators (CELMoDs), a novel class of immunomodulatory agents showing promising efficacy in MM [113]. Despite these options, resistance to BCMA-directed therapies—via antigen loss or T-cell exhaustion—is emerging [114]. The sequencing of BCMA therapies (CAR T, bispecifics, ADCs) is still being optimized in clinical trials [115].

Resistance to anti-CD38 agents can also develop over time, and the utility of re-treatment strategies or alternative combinations is under investigation [116]. Monitoring MRD has become an important tool to assess the depth of treatment response, as MRD negativity is associated with improved progression-free and overall survival [111].

Overall, these advancements have reshaped the therapeutic landscape of MM, enabling clinicians to tailor treatment more precisely and improve patient outcomes [112].

### 3.7. Comparative Overview of Precision Medicine Targets in Hematologic Malignancies

To summarize the molecular targets and strategies discussed above, Table 2 presents a comparative overview across major hematologic malignancies.

## 4. Emerging Concepts and Innovations

The field of precision medicine in hematological malignancies is rapidly evolving, with several innovative technologies on the horizon. These advancements hold the potential to further refine our understanding of disease biology and personalize treatment strategies. Importantly, translating these technologies into clinical practice requires not only scientific rigor but also practical frameworks for integration, such as standardized protocols, clinical-grade platforms, and collaboration across molecular pathology and oncology teams [117].

### 4.1. Single-Cell Sequencing

Traditional bulk sequencing technologies capture the average molecular profile of a cell population, often masking the inherent heterogeneity within a tumor. Single-cell sequencing fills this gap by analyzing the genomic, transcriptomic, or epigenomic profiles of individual cells [118]. In hematologic malignancies, this method provides useful insights into tumor diversity, including clonal evolution and interactions between cancerous cells and their microenvironment [119].

For instance, in AML, single-cell RNA sequencing (scRNA-seq) has identified leukemic stem cell subpopulations associated with chemoresistance and relapse, guiding the development of combination therapies that target resistant clones [120]. In cutaneous T-cell lymphoma, single-cell approaches have distinguished between malignant and reactive T-cell populations, improving diagnostic accuracy and reducing the need for invasive procedures [121].

Clinical integration is emerging via academic–industry partnerships. Some cancer centers now use single-cell profiling during relapse workups to identify resistant subclones and tailor salvage therapies. Moreover, platforms like Mission Bio’s Tapestri are being adapted for clinical use, enabling simultaneous single-cell DNA and protein analysis [122].

These insights are critical for guiding the development of targeted therapies that address cellular heterogeneity [119]. Nonetheless, single-cell sequencing is limited by challenges such as computational and bioinformatics complexity [123].

### 4.2. Spatial Transcriptomics

Single-cell sequencing offers detailed molecular insights at the cellular level but misses the spatial arrangement of cells within tissues. Spatial transcriptomics overcomes this by capturing gene expression profiles while preserving cellular organization in tissue samples [124]. This approach offers a comprehensive view of tissue architecture, enhancing our understanding of cellular interactions and niche-specific expression, especially in lymphomas and leukemias, where the microenvironment plays a crucial role [125,126].

Practical applications include mapping immune evasion zones and treatment-resistant niches. For example, spatial transcriptomics has been used to visualize immune exclusion in DLBCL, identifying tumor regions with reduced cytotoxic T-cell infiltration [127]. This has implications for selecting patients who may benefit from immune checkpoint blockade or local immune-modulatory interventions [128].

Clinical integration is underway in translational settings. Some centers now pair spatial data with histopathology and immunohistochemistry to refine diagnoses or predict response to immunotherapies [129]. Companies like 10x Genomics and NanoString have developed user-friendly spatial platforms compatible with the FFPE tissue, allowing more routine use in pathology labs [130]. Future clinical trials may stratify patients based on spatial immune profiles to guide combination immunotherapies [129].

It can identify functional regions like immune-excluded zones and reveal how proximity to immune or stromal cells influences disease progression and treatment response, aiding in biomarker discovery and therapeutic targeting [125,131].

### 4.3. Drug Repurposing

Drug repurposing offers a faster and more cost-effective alternative to traditional drug development by identifying new therapeutic uses for existing medications originally approved for other conditions [132]. In hematologic malignancies, this strategy accelerates the discovery of treatments by employing known pharmacology and safety profiles. Through computational screening and molecular profiling, researchers can identify drugs with activity against specific cancer-related targets or pathways [133,134]. This is especially valuable for rare or treatment-resistant cancers, where therapeutic options are limited [134,135]. Notable examples include antidiabetic, anti-inflammatory, and antiparasitic agents demonstrating anti-cancer effects in preclinical studies.

For example, metformin, commonly used in type 2 diabetes, has demonstrated anti-leukemic effects in CML by inhibiting mTOR signaling, and is now under investigation in early-phase trials as an adjuvant therapy. Similarly, acetylsalicylic acid (aspirin) has shown preclinical efficacy in multiple myeloma by suppressing NF-κB and ERK signaling pathways, which are often dysregulated in MM [133].

In a more advanced clinical example, thalidomide, initially used as a sedative, was repurposed for multiple myeloma and eventually led to the development of modern immunomodulatory drugs (IMiDs) like lenalidomide [136]. Ongoing drug repurposing screens using computational tools (e.g., Connectivity Map, DrugBank) are identifying agents like antiparasitic drugs (e.g., ivermectin) and antihistamines with selective cytotoxicity against leukemia cells [133,137,138].

## 5. Clinical Implementation and Challenges

Despite the pioneering potential of precision medicine in hematologic malignancies, several challenges limit its widespread adoption in routine clinical practice.

### 5.1. High Cost of Genomic Testing

Advanced molecular testing, particularly NGS, remains costly and is not uniformly reimbursed by healthcare systems [139]. The expense can be prohibitive for many institutions, especially in low- and middle-income countries, limiting the reach of precision medicine [140]. Even in high-income settings, insurance coverage may not extend to comprehensive genomic panels unless specific clinical criteria are met, further restricting patient access [141].

### 5.2. Access Disparities

Geographic and socioeconomic factors significantly influence access to precision diagnostics and targeted therapies. Patients in rural or under-resourced areas often lack access to healthcare facilities with molecular diagnostic services. Similarly, disparities in healthcare infrastructure and provider training can lead to the unequal implementation of genomic technologies, widening the gap in outcomes between different patient populations [140,142]. A notable example is Nigeria, where the HER2-targeted therapy trastuzumab became available only five years after its approval in the United States and Europe, highlighting the significant delay in access to life-saving cancer treatments between high-income and low- and middle-income countries [140].

### 5.3. Lack of Standardization

There is considerable variability in how genomic testing is conducted, interpreted, and reported [21]. Differences in panel composition, sequencing depth, and bioinformatic pipelines can lead to inconsistencies in clinical decision making [143,144]. Although guidelines from expert groups like the Association for Molecular Pathology (AMP), the American Society of Clinical Oncology (ASCO), and the College of American Pathologists (CAP) exist for interpreting somatic mutations, they differ in how they define a variant’s cancer-causing potential. This lack of consistent standards complicates the use of molecular findings in guiding treatment decisions for hematologic cancers [145].

### 5.4. Integration into Clinical Workflows

The integration of molecular data into clinical decision making remains a challenge [146]. Interpreting complex genomic reports requires multidisciplinary collaboration among hematologists, molecular pathologists, bioinformaticians, and genetic counselors [147]. Moreover, existing electronic health record (EHR) systems are often ill equipped to store or analyze complex molecular data, creating barriers to smooth incorporation into patient care [148].

Overcoming these challenges will require coordinated efforts to reduce costs, expand access, standardize testing protocols, and build the necessary infrastructure and workforce to support precision medicine. Addressing these barriers is essential to ensure that the benefits of precision hematology are equitably attained in diverse healthcare settings [139,142,149].

## 6. Future Directions

As precision hematology continues to evolve, multiple promising technologies are set to reshape diagnostics, treatment, and prevention. These innovations improve not only molecular profiling but also the integration of immune profiling, predictive analytics, and preventive strategies into hematologic care.

### 6.1. Pan-Cancer Trials Based on Molecular Targets

Traditional clinical trials have historically focused on specific diseases, often ignoring shared molecular alterations between different hematologic cancers. However, the emergence of “pan-cancer” basket trials aims to enroll patients based on actionable genetic alterations rather than tumor lineage and pathology. Such trials promote drug repurposing and accelerate therapeutic discovery for rare malignancies with limited treatment options [150,151].

### 6.2. Personalized Immunotherapy

The integration of immunotherapy into hematologic malignancy treatment, such as CAR T-cell therapy, BiTEs, and immune checkpoint inhibitors, has been transformative [152]. Future directions include tailoring these approaches to individual patient immune profiles using high-throughput immune repertoire sequencing (to analyze the diversity of immune cells), neoantigen prediction (to identify tumor-specific antigens that can be targeted by the immune system), and single-cell analysis [153,154,155]. Personalized immunotherapies may improve efficacy while reducing toxicities, moving from broad treatment regimens to individualized immune modulation [156].

### 6.3. Precision Prevention

While precision medicine often focuses on treatment, prevention remains an essential yet often overlooked area of care [157]. Advances in germline genetics and polygenic risk scoring can help identify individuals at an elevated risk of hematologic malignancies [158,159]. Inherited predisposition syndromes (e.g., Li–Fraumeni, Fanconi anemia, GATA2 deficiency) as well as somatic conditions like clonal hematopoiesis of indeterminate potential (CHIP) offer opportunities for early surveillance or preventive interventions [160,161,162,163]. The development of precision prevention strategies could ultimately reduce disease burden through earlier detection and risk mitigation [164].

### 6.4. AI-Powered Treatment Algorithms

Artificial intelligence (AI) and machine learning (ML) are increasingly applied in hematology to analyze complex multi-omic data, including genomic, transcriptomic, proteomic, imaging, and clinical information, to uncover patterns and guide personalized treatment. These algorithms integrate data such as genetic profiles, lab results, and patient histories to improve prognosis accuracy, stratify patients, predict therapy response or relapse, and support real-time, data-driven decision making. As they continuously learn from real-world inputs, AI-powered tools hold promise to optimize therapeutic strategies and improve outcomes in hematologic malignancies [165,166].

## 7. Conclusions

Precision medicine has revolutionized hematologic cancer care by tailoring therapies to individual molecular and clinical profiles. Driven by advances in genomic, epigenomic, proteomic, and liquid biopsy technologies, this approach has significantly improved diagnosis, risk stratification, and treatment strategies across leukemias, lymphomas, and multiple myeloma. Despite challenges related to cost, accessibility, and clinical implementation, emerging innovations—including single-cell profiling, artificial intelligence, and personalized immunotherapies—promise to deepen our understanding of disease biology and pave the way for more effective and individualized care. Ensuring equitable integration of these advances into routine practice will be essential for achieving the full potential of precision hematology.

## Figures and Tables

**Table 1:** Comparison of genomic profiling approaches in precision hematology.

	NGS	WES	RNA-Seq
**Coverage**	Whole genome or targeted (coding + non-coding)	Protein-coding regions (~1% of genome)	Transcriptome (expression, fusions)
**Main applications**	Mutation profiling, MRD, therapy guidance	Somatic/germline mutations detection	Fusion detection, expression profiling, pathway/splicing analysis
**Depth and sensitivity**	High, detects low-frequency variants	Moderate, may miss rare variants	Expression-dependent, RNA-quality-sensitive
**Strengths**	Broad variant detection, clinically actionable	Efficient for coding variants, useful in rare cases	Functional insights, detects fusions missed by DNA sequencing
**Limitations**	Cost, complex data, bioinformatics challenges, standardization gaps	Misses non-coding/structural variants; lower depth	RNA degradation risk; limited clinical adoption (but growing)

NGS: next-generation sequencing; WES: whole-exome sequencing; RNA-seq: RNA sequencing; MRD: minimal residual disease.

**Table 2:** Examples of precision medicine targets across hematologic malignancies.

Malignancy	Molecular Targets/Biomarkers	Targeted Therapy
AML	*FLT3*, *IDH1/2*, *NPM1*, *TP53*	FLT3 inhibitors (midostaurin, gilteritinib, quizartinib); IDH inhibitors (ivosidenib, enasidenib); hypomethylating agents (azacitidine, decitabine); investigational: APR-246 (eprenetapopt); menin inhibitors (revumenib, ziftomenib)
ALL (B-cell)	*BCR-ABL1* (Ph+), *ABL-class/JAK* fusions (Ph-like), CD19, CD22	TKIs (imatinib, dasatinib, asciminib, olverembatinib, ponatinib); BiTE (blinatumomab); CAR T (tisagenlecleucel, KTE-X19 [brexucabtagene autoleucel]); ADC (inotuzumab ozogamicin)
ALL (T-cell)	*NOTCH1*, *CDK4/6*, *BCL-2*	Investigational: venetoclax, navitoclax, NOTCH1 inhibitors, CDK4/6 inhibitors
CML	*BCR-ABL1*, *T315I* mutation	TKIs (imatinib, dasatinib, nilotinib, bosutinib, ponatinib, asciminib)
CLL	*TP53*, *del(17p)*, *IGHV* mutation status, *NOTCH1*, *SF3B1*, *BIRC3*	BTK inhibitors (ibrutinib, acalabrutinib, zanubrutinib); BCL-2 inhibitor: venetoclax ± Obinutuzumab; Emerging: pirtobrutinib
Lymphomas	*MYC/BCL2/BCL6* rearrangements, *PD-L1* (9p24.1)	BTK inhibitor: ibrutinib (ABC-DLBCL); immune checkpoint inhibitors (nivolumab, pembrolizumab); ADC: polatuzumab vedotin; anti-CD19 (tafasitamab + lenalidomide); selinexor; CAR T (axi-cel, liso-cel, tisa-cel)
Multiple Myeloma	*t(4;14)*, *t(14;16)*, *del(17p)*, BCMA	BCMA-targeted CAR T-cells (idecabtagene vicleucel, ciltacabtagene autoleucel), bispecifics (teclistamab, elranatamab), ADCs (belantamab mafodotin), CELMoDs (e.g., iberdomide, mezigdomide), anti-CD38 antibodies (daratumumab, isatuximab)

AML: acute myeloid leukemia; ALL: acute lymphoblastic leukemia; CML: chronic myeloid leukemia; CLL: chronic lymphocytic leukemia; CAR T: chimeric antigen receptor T-cell therapy; BiTE: bispecific T-cell engager; ADC: antibody–drug conjugate; TKI: tyrosine kinase inhibitor; BTK: Bruton’s tyrosine kinase; ABC-DLBCL: activated B-cell-like diffuse large B-cell lymphoma; Ph+: Philadelphia chromosome-positive; Ph-like: Philadelphia chromosome-like; BCMA: B-cell maturation antigen; CELMoDs: cereblon E3 ligase modulators.

## Data Availability

No new data were created or analyzed in this study.
