# Correction: Khoury et al. Precision Medicine in Hematologic Malignancies: Evolving Concepts and Clinical Applications. *Biomedicines* 2025, *13*, 1654

**DOI:** 10.3390/biomedicines14081793

**Published:** 2026-08-10

**Authors:** Rita Khoury, Chris Raffoul, Christina Khater, Colette Hanna

**Affiliations:** 1 Division of Hematology/Oncology, Lebanese American University Medical Center-Rizk Hospital, Beirut 1100, Lebanon; rita.khoury011@gmail.com (R.K.); christina.khater01@lau.edu.lb (C.K.); colette.hanna@lau.edu.lb (C.H.); 2 Department of Internal Medicine at VCU Health, Richmond, VA 23298, USA

**References**

As Ref. 165 was retracted, it has been deleted from the paper [1]. With this correction, the order of some references has been adjusted accordingly. The authors state that the scientific conclusions are unaffected. This correction was approved by the Academic Editor. The original publication has also been updated.
